# Experimental Prognostication of Ultra-High-Performance Lightweight Hybrid Fiber-Reinforced Concrete by Using Sintered Fly Ash Aggregate, Palm Oil Shell Aggregate, and Supplementary Cementitious Materials

**DOI:** 10.3390/ma15145051

**Published:** 2022-07-20

**Authors:** Diptikar Behera, Kuang-Yen Liu, Dineshkumar Gopalakrishnan

**Affiliations:** 1 Department of Civil Engineering, National Cheng Kung University, Tainan 701, Taiwan; 2 Department of Civil Engineering, Vaagdevi College of Engineering, Warangal 506005, India; gdkcivil@gmail.com

**Keywords:** reinforced lightweight concrete, hybrid fiber, silica materials, XRD, SEM, EDS, ANOVA, regression

## Abstract

To create cost-effective structures, the modern construction industry has sought to reduce the dead load of buildings. Lightweight concrete is a quick way to reduce dead load. The current study is primarily concerned with identifying modern substitutes for coarse aggregate likely to aid in waste management and offer potential alternatives to the most exploited natural resources. According to ACI C 39-M, this study developed a novel lightweight hybrid fiber-reinforced concrete (LWHFRC) with a density of less than 1825 kg/m^3^ and compressive strength of 50 to 75 MPa. Ordinary Portland cement (53 Grade) was mixed with fly ash, silica fume, and GGBS. Sintered fly ash aggregate (SFA) and palm oil shell aggregate (POS) were used as coarse aggregates. Hooked steel fibers and polyvinyl alcohol fibers were combined in a hybrid form to improve crack propagation properties at the initial and subsequent stages. The water-to-binder ratio was kept constant at 0.30 to 0.35 with a 1% superplasticizer. Four volume fractions of hybrid fibers (both steel and PVA with Vf = 0%, 1%, 1.5%, and 2%) were added. In addition, XRD, SEM, EDS, and EDS mapping tests were performed to finalize the material’s chemical composition and crystalline structure. Furthermore, beams and cylinders were tested to determine the modulus of rupture, which was determined to be between 9.5 and 14 MPa by ACI code C 1609-M, and indirect tensile strength, achieved as 10 to 14 MPa by ACI code C 496-M. The researcher altered the modulus of elasticity (Ec) formula for lightweight concrete and discovered a relationship between fc’ and fcb, fc’ and fspt, and fcb and fspt. Finally, ANOVA and regression tests were run to check the significance of the experiment. The cost analysis revealed that the cost of LWHFRC increased by approximately 16.46%, while the strength increased by 55.98% compared to regular concrete.

## 1. Introduction

Advancements in expertise enhance not only human comforts but also impairment the environment. In the world, most industries are accompanied by many materials such as fly ash [1,2,3,4], silica fume, and GGBS. As a natural vegetable aggregate, palm oil shell is a vegetable product from palm trees with low density that satisfies lightweight coarse aggregate criteria. As an artificial aggregate, sintered fly ash aggregate with low density and good strength is also an artificial aggregate for the palletization process in industries [5,6]. Palletization is a worldwide process used to manufacture artificial aggregates named sintered fly ash aggregate [7]. Fly ash is a by-product of coal-based thermal power plants. If not properly disposed of, fly ash can cause water and soil contamination, consequently interrupting the ecological cycles. China, the USA, and India consume around 70% of the total coal worldwide [8]. According to a CEA [9] report, about 166 million tons of fly ash in India are generated from 132 thermal plants annually. About 56% of fly ash is utilized effectively through various methods, and the remaining fly ash is still a concern to society. Most attention is devoted to commercial applications such as the replacement of cement. This process simultaneously consumes the generated industrial wastes and reduces the requirement for cement clinker. High-quality fly ash with a low carbon content is used as a mineral additive to manufacture cement and concrete. Low-quality fly ash with higher and variable carbon content is generally used in landfills. Production of artificial aggregates from fly ash [10] is a great leap toward fly ash disposal in large quantities. Generally, the aggregate phase occupies 60–80% of the concrete matrix by volume. The depletion of natural aggregate resources is another significant concern for sustainable development worldwide [11].

Steel fiber and polyvinyl alcoholic fiber (PVA) were also used independently and combined to produce high-performance fiber-reinforced lightweight concrete by a few researchers. The researcher’s primary goal is to use an environmentally harmful material as a light component to create high-performance, lightweight concrete. However, few researchers have already developed ultra-high-performance fiber-reinforced lightweight concrete, which is most suitable for structural members such as beams, columns, floors, and precast members. However, this was very expensive in the concrete industry because most researchers did not use coarse aggregate in their ultra-high-performance concrete (UHPC). In addition, many researchers developed only lightweight concrete. We plan to create new inventive concrete that will be high-performance and lightweight with truncated cost. There are numerous benefits of lightweight structural concrete over normal-weight concrete in the construction industry, particularly in high-rise buildings. The original method of fabricating lightweight structural concrete uses lightweight aggregates instead of ordinary aggregates in concrete. Due to the insufficient resources for natural and artificial lightweight aggregates, the substitute sources for lightweight aggregates should be revealed from industrial wastes. Oil palm shell (OPS) and oil palm-boiler clinker (OPBC) are two solid wastes from the palm oil industry [12,13] and are obtainable in abundance in tropical regimes. In this paper, the effects of using fly ash (FA), silica fume (SF), ground granulated blast slag (GGBS), [14] and numerous combinations of them such as sintered fly ash aggregate (regular round shape) and palm oil shell aggregate (irregular triangular shape) [15] with two different fibers such as steel fiber and PVA fiber were evaluated. Another goal of the researcher was to investigate multiaggregate (SFA and POS) without natural coarse aggregate (NCA) because circular SFA [16] and triangular POS have different workability and water absorption criteria for maintaining the perfect bonding of lightweight concrete [17] in construction industries. The effect of fibers on mortars and concrete has recently been demonstrated to increase the ductility chattels of cementitious composites.

Superior tensile strength, flexibility, toughness, and crack resistance were proven as the main properties enhanced by fiber reinforcement. Hybrid fiber-reinforced composites were renowned for further improving the properties at the early and future stages of crack and propagation. The key is to combine various beneficial properties of fiber into a single matrix, resulting in superiority in both aspects. Steel fiber and PVA fiber [18,19] were combined with two lightweight aggregates, palm oil shell, sintered fly ash aggregate, and three mineral admixtures, fly ash, silica fume, and GGBS in this study. The first series is industrialized with the optimum mineral admixture in different percentages, while the second series is settled with the lightweight concrete [20] with optimum mineral admixture. Finally, the third series explores the optimum lightweight concrete [21,22] with different percentages of two fibers [23]. The most crucial aspect of this research was maintaining the W/B ratio of 0.30 and 1% of high-range water-reducing admixture (HRWRA) throughout all three series. The compressive strength, flexural strength, split tensile strength, water absorption test, workability test, XRD test, SEM test, EDS test, and EDS mapping were carried out in the first stage of the research. The second stage of the study is to perform the bond shear test (slant shear and pull out) of lightweight concrete with regular concrete for the retrofitting field of the construction industries. In addition, the researcher plans to perform different durability tests for the new LWHFRC. After completing the material level of the research, the researcher will test other beam loadings to satisfy the new mix design concrete for high-performance, lightweight concrete. This paper investigates the provision of two different fibers, steel, and PVA fiber, in hybrid form with multi-lightweight aggregate by adding all three mineral admixtures to develop high-strength and high-performance concrete. Analysis shows that performance enhancement was obtained by using fibers in the hybrid form. Hooked steel fibers with an aspect ratio of 40 and 80 and PVA fibers [24] with an aspect ratio of 800 were used in this research. The size of the normal coarse aggregate (NCA), sintered fly ash aggregate (SFA) of size 12.5 to 18 mm, and palm oil shell aggregate (POS) of 10 to 12.5 mm were used. This study achieved better lightweight concrete strength and high-performance hybrid fiber-reinforced concrete. The cost of lightweight concrete [25] was also reduced in this research. According to the cost analysis, the cost of LWHFRC increased by approximately 16.46%, while the strength increased by 55.98% compared to regular concrete in the same mix design without mineral admixture.

The mix design is based upon ACI-213 R-03 and ACI-211.2. In this research, 20 to 50% SFA and 10 to 30% POS gave better results. The higher volume of GGBS has a tremendous role in the high strength of concrete. Finally, ANOVA and regression analyses were performed to identify the lightweight hybrid fiber-reinforced [26,27,28,29] concrete (LWHFRC). The researcher modified the modulus of elasticity (Ec) with a compressive strength (fc’) formula for lightweight concrete and created a relationship equation between compressive strength and flexural strength (fc’ and fcb), compressive strength and split tensile strength (fc’ and fspt), flexural strength [30], and split tensile strength [31] (fcb and fspt) (ACI-318-05). The forthcoming paper will discuss hybrid fiber-reinforced lightweight concrete’s bond strength and durability. In the next research phase, beam structural behavior parameters such as load-carrying capacity, ductility factor, stiffness, energy absorption capacity, and energy index will be evaluated.

The development of lightweight concrete without sacrificing performance or strength is a novel aspect of this study. This novel material alters design considerations and reduces the self-weight of the structure. This study will cover the numerous practical applications in civil engineering practices. Because of their overwhelming strength and performance, these innovative concrete composites may be used in earthquake-prone areas. Because sintered fly ash aggregate (SFA) and palm oil shell aggregate (POS) have regular and irregular shapes in structure, researchers had the brilliant idea of combining both totals with a high volume of cementitious materials without natural aggregate to achieve lightweight, high-performance concrete at a lower cost for bridge structures and high-rise building applications.

## 2. Materials and Methods

**Figure 1:**
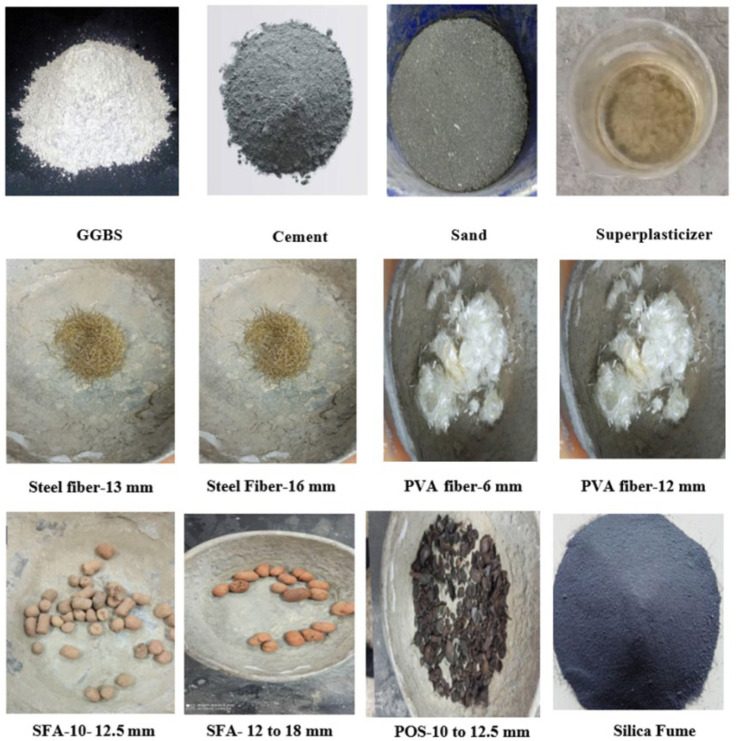
Raw materials.

**Figure 2:**
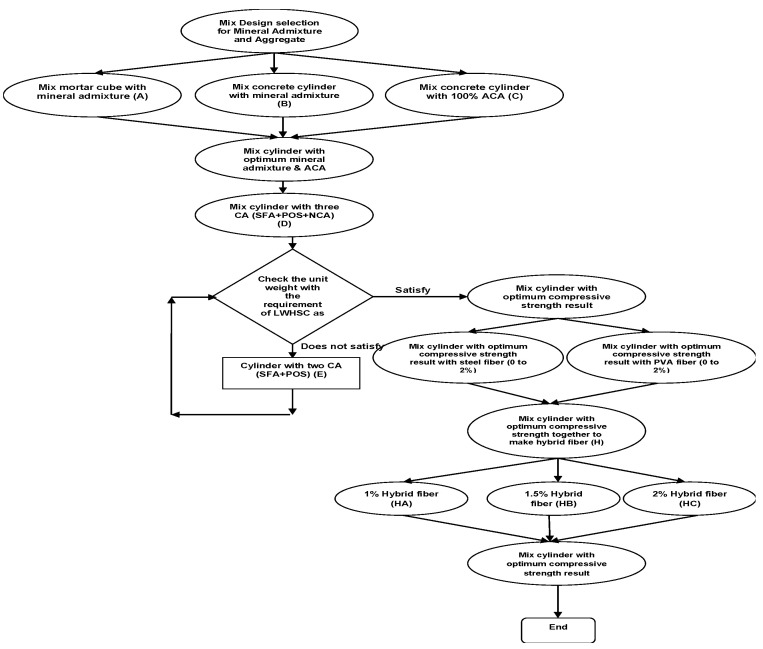
Flow chart of primary mix design.

Twenty-four mix designs with varying volumes of steel and PVA fibers were prepared to investigate the effect of steel and PVA fibers on the properties of lightweight aggregate concrete. The volume fraction of steel fibers and PVA fibers in concrete ranged from 0% to 2%. Figure 1 depicts the raw materials with sizes. Figure 2 summarizes the fiber contents of all 18 assorted designs. All other parameters, such as cement content (c = 600 kg/m^3^), water/cement ratio (w/c = 0.3 to 0.35), superplasticizer dosage (SP = 1%), and weight ratio of coarse lightweight aggregate to natural river sand (LWA/S = 1.5), remained constant across all mixtures. The water absorption of aggregates was determined within the mixing time, and the batch proportions were adjusted accordingly.

### 2.1. Material Properties

The properties of the materials are listed in Table 1 below. In this mix, two mineral admixtures with 10% silica fume and 35% GGBS by partial replacement of cement, two coarse aggregates such as SFA and POS without NCA, and two fibers such as steel fibers and PVA fibers of 0 to 2% fraction by volume to make hybrid fiber-reinforced concrete, 0.30 to 0.35 W/B ratio, with high volume cementitious materials around 600 kg/m^3^ are used. 

### 2.2. Mix Proportion

The 24-mix design (E1 to E6, HA1 to Ha6, HB1 to HB6, and HC1 to HC6) used in this experiment is as follows. The quantity of the materials used in this experiment was calculated from that mixed design. The number of specimens in this experiment is 576 pieces divided into four series. All mixing and preparation of samples were performed at room temperature. The first series consists of a cylinder specimen (100 mm × 200 mm) for compressive strength. The second series consists of the flexural specimen (150 mm × 150 mm × 530 mm) for flexural strength, and the third series consists of a cylinder specimen (150 mm × 300 mm) for the split tensile strength of concrete. The four series consists of a cylinder specimen (100 mm × 200 mm) for the water absorption test. The volume fraction (Vf) of both the steel fiber and PVA fiber was also categorized in a hybrid form of 1% (HA series), 1.5% (HB Series), and 2% (HC Series). The amount of each material used in this experiment is shown in Table 2 and Table 3. All the materials shown in Table 2 and Table 3 are magnified with a 1.3 amplification factor for casting. According to the flow chart given in Figure 2, we decided on the mix design by ACI code 213 R-03 and ACI-211.2. The flow chart in Figure 2 depicts the complete summary of our research work mix design stage. The results of hybrid fiber-reinforced lightweight concrete (HA, HB, and HC Series) were only highlighted in this paper. In another article, the researcher attached the other results of individual fiber-reinforced lightweight concrete.

The fine and coarse aggregates were mixed in a concrete mixer for about 2 min before cement was added to the concrete mixtures. The ingredients were re-mixed until a uniform color was achieved. The steel fiber and PVA fiber were then gradually added to the mixture. The water had been split in half. The first half was added to the ingredients in the mixer and mixed for 2–3 min before adding the remaining water and superplasticizer. After that, the ingredients were mixed for another 2–3 min. The concrete was then poured into the molds and consolidated on a vibrator. The specimens were cured under laboratory conditions by covering them with a plastic sheet for 24 h, followed by wet curing. Figure 2 depicts a summary of casting details. Table 2 and Table 3 also include a mix design summary for hybrid fiber-reinforced concrete and lightweight concrete.

### 2.3. Evaluation

The effect of partial/complete substitution of coarse artificial aggregate with POS on mechanical properties such as unit weight, compressive strength, flexural strength, split tensile strength, modulus of elasticity, water absorption test, and workability test was evaluated (slump cone).

#### 2.3.1. Unit Weight

After 28 days of curing, three cylindrical concrete specimens measuring 100 mm in diameter and 200 mm in height were prepared from each concrete mixture to determine the unit weight. The specimens were air-dried for 48 h before being measured to ensure uniform moisture content. The weight-to-volume ratio was expressed as the unit weight of concrete. For each mixture, triplicate measurements were taken, and the average values were reported. Table 2 and Table 3 provide information on unit weight.

#### 2.3.2. Workability Test

Workability tests such as slump, compaction factor, and vee bee consistometer were performed on various lightweight concrete and lightweight hybrid fiber-reinforced concrete mixtures. Table 4 displays the results. A sulfated naphthalene-based superplasticizer was used in additional trials to improve the workability and cohesiveness of fresh concrete [32]. A superplasticizer content of 1% by mass of binder was used.

#### 2.3.3. Slump Test

The slump test was carried out following ACI-C143/C143M 12. A 300 mm high frustum of a cone with a leveled surface was placed in the mold. The concrete was filled in three layers, with each layer tamped 25 times with a 16 mm steel rod with a rounded nose. The top was not level, and the mold was firmly pressed against the slab base. The mold was gently lifted, and the decrease in concrete height was measured and recorded in Table 4 [32].

#### 2.3.4. Compaction Factor Test

The compacting factor test was performed following ACI-C143/C143M-12 using a compacting factor test apparatus. The top hopper was filled gently with concrete, while the bottom hopper was kept closed. The top hopper was opened, and the concrete fell from the upper to the lower hopper and then from the lower hopper to the mold. Table 4 summarizes the test values. The density of partially compacted concrete was divided by the density of fully compacted concrete to determine the degree of compaction.

#### 2.3.5. Vee Bee Time Test

The vee bee time apparatus measured the workability of concrete as per ACI-C143/C143M-12. The time required for the complete remolding of concrete was measured. The vee bee times are tabulated in Table 4 [32].

#### 2.3.6. Water Absorption Test

At 28 days of curing, the cylinder samples were tested for water absorption capacity. First, the dry masses of concrete specimens were saturated in the water basin for 28 days in the methodology. Following that, the saturated concrete samples were reweighed, and the soaked mass of the samples was subtracted from the dry mass to determine the mass of water absorption and thus the percentage of water absorption relative to dry mass. The rates of water absorption by the sample cylinder specimen are shown in Table 4 [32].

#### 2.3.7. Cylinder Compressive Strength Test

The cylinder compressive strength test was performed on the 100 mm diameter and 200 mm high cylinder specimens at the ages of 7 and 28 days, respectively, using a 100-ton capacity compression testing machine following ACI-C39/C39M-20 [32].

#### 2.3.8. Split Tensile Strength Test

The split tensile strength test is an indirect tensile strength test for cylindrical specimens. Splitting tensile strength tests were performed at 28 days for concrete cylinder specifications of 150 mm in diameter and 300 mm in length, using an ASTM-C496 [33]. Figure 6 depicts the test specimen. The load was applied gradually until the specimen split and readings were noted.

The splitting tensile strength was estimated after 28 days by using the following relationship:

f_spt_ = 2P/πld

where

f_spt_ = splitting tensile strength of the specimen (MPa);
P = maximum load applied to specimen (N).
l = length of the specimen (mm).
d = cross-sectional diameter of the specimen (mm).

#### 2.3.9. Flexural Strength Test

A flexural strength test was performed on the 150 mm × 150 mm × 530 mm beam specimen at 28 days using a 50-ton capacity universal testing machine by subjecting the specimen to four-point bending to determine the flexural strength as per ASTM C 1609 M. The flexural strength was calculated using the result analysis and test configuration formula. 

## 3. Results and Discussion

### 3.1. Unit Weight

As a substitute for SFA, the unit weight of concrete specimens prepared with varying amounts of POS is used. In Table 2 and Table 3, these values ranged from 1819 to 2487 kg/m^3^. The unit weight of concrete from the C1 to C3 series was used to replace NCA, SFA, and POS completely. However, the unit weights of C2 and C3 fell within the ACI 213 R-03 lightweight concrete criteria. The E1 to E6 series were also within the light concrete unit weight range of 1819–1828 kg/m^3^.

### 3.2. Workability and Water Absorption Test

**Figure 3:**
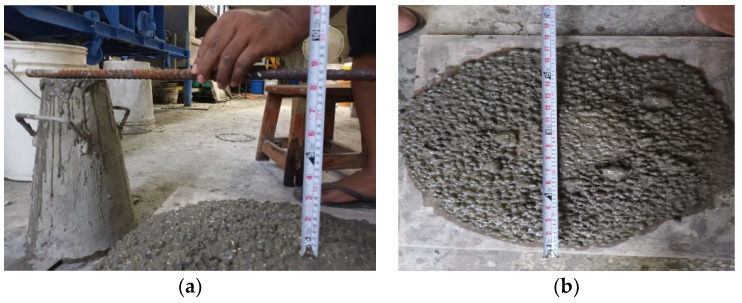
Slump cone test (height = 24.2 cm and slump flow diameter = 51 cm).

Fresh concrete is usable when it can be easily transported, placed, compacted, and finished without segregation. Slump tests were performed on concrete with lightweight aggregate from E1 to E6 as lightweight concrete (LWC) and HA to HC series as lightweight hybrid fiber-reinforced concrete (LWHFRC) to determine comparable workability. Before the fresh concrete specimens were cast in the molds, four batches of all concrete types were tested for workability. Table 4 displays the test results for the average slump of control lightweight concrete and all LWHFRC concretes. The average slump flow diameter (E3—without fiber) was 51 cm, with a slump height of 24.2 cm, as shown in Figure 3a and Figure 3b, respectively. 

At 28 days of curing, the cylinder samples were tested for water absorption capacity. First, the dry masses of concrete specimens were saturated in a water basin for 28 days. Following that, the saturated concrete samples were reweighed. Finally, the soaked mass of the samples was subtracted from the dry mass to determine the mass of water absorption and, thus, the percentage of water absorption relative to dry mass. Table 4 shows the rates of water absorption by a cylinder specimen sample. Because of the hydrophilic nature of fiber and the larger interfacial area between the fiber and the matrix, water absorption increased gradually as fiber content increased.

### 3.3. Compressive Strength

(a) E Series (LWC)

**Figure 4:**
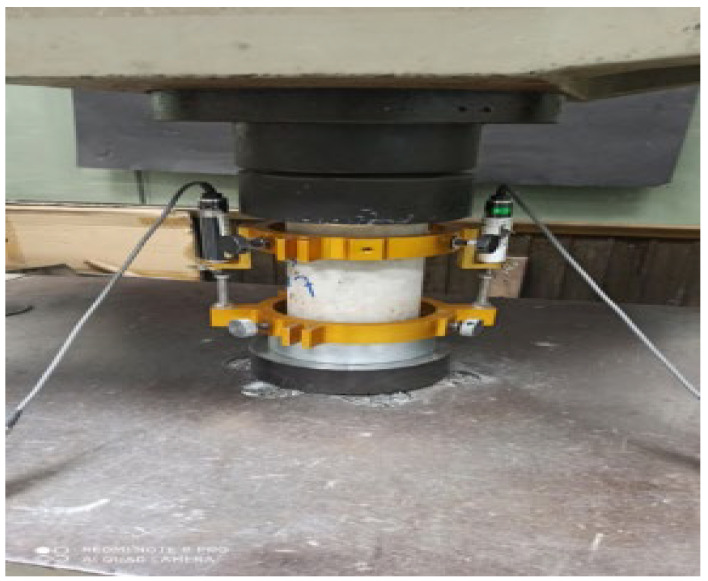
Cylinder compressive strength test with LVDT.

**Figure 5:**
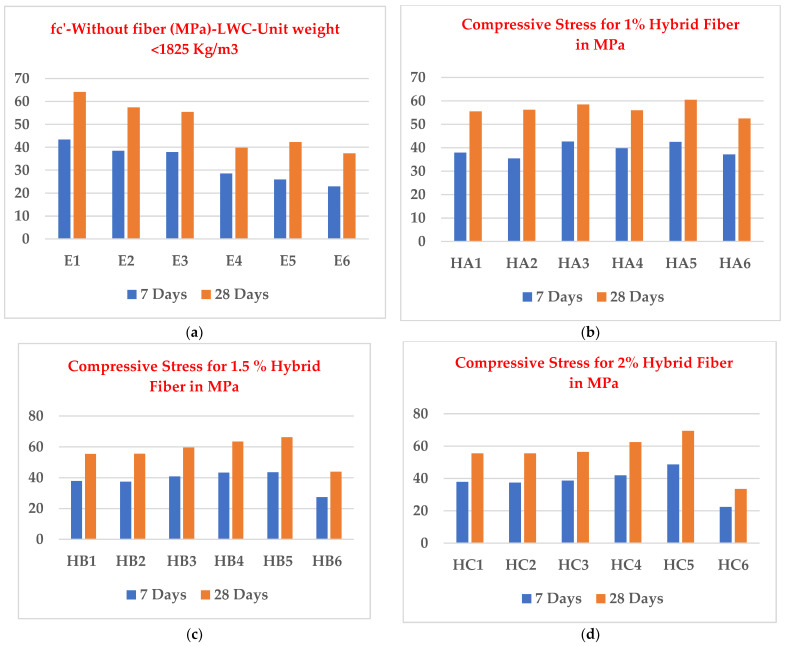
Compressive strength of concrete with different fiber contents.

Table 5 shows the cylinder compressive strength of LWC (lightweight concrete) and LWHFRC (lightweight hybrid fiber-reinforced concrete) at 7 and 28 days. At 28 days, the compressive strengths (E1-E6) of LWC were increased by 80.44%, 61.53%, 55.99%, 11.83%, 18.78%, and 4.93%, respectively, when compared to control concrete (C1). At 28 days, the compressive strength of 100% SFA (E1) was 64.145 MPa, nearly as high as the other results in this series. The scores for 90% SFA + 10% POS (E2) and 80% SFA + 20% POS (E3) were 57.425 and 55.453, respectively. So, for this next stage, the researcher used the E3 series to keep the unit weight below the lightweight criteria (below 1850 kg/m^3^) set by ACI-213 R-03 and developed a strong bond between irregular triangular coarse aggregate (POS) and regular circular coarse aggregate (SFA) at a 1:4 ratio (POS: SFA). Figure 4 shows the cylinder compressive strength test with LVDT. Figure 5a graph shows the compressive strength of concrete without fiber.

(b) HA Series (1% Hybrid Fiber)

At the age of 28 days, the compressive strengths (HA1-HA6) of LWHFRC (1% hybrid fiber) were increased by 55.99%, 57.96%, 64.44%, 57.48%, 69.97%, and 47.50%, respectively, when compared to control concrete (C1). At 28 days, the compressive strength of 1% steel fiber (HA5) was 60.425 MPa, nearly as high as the other results. Figure 5b graph shows the compressive strength of concrete with 1% hybrid fiber.

(c) HB Series (1.5 % Hybrid)

At the age of 28 days, the compressive strengths (HB1-HB6) of LWHFRC (1.5% hybrid fiber) were increased by 55.99%, 56.24%, 67.59%, 78.58%, 86.34%, and 23.42%, respectively, when compared to control concrete (C1). At 28 days, the compressive strength of 1.5% steel fiber (HB5) was 66.245 MPa, nearly as high as the other results in this series. Figure 5c graph shows the compressive strength of concrete with 1.5% hybrid fiber.

(d) HC Series (2% Hybrid)

At the age of 28 days, the compressive strengths (HC1-HC6) of LWHFRC (2% hybrid fiber) were increased by 55.99%, 56.24%, 58.81%, 75.63%, 95.29%, and 5.85% (-ve), respectively, when compared to control concrete (C1). At 28 days, the compressive strength of 2% steel fiber (HC5) was 69.425 MPa, nearly as high as other results in this series. Figure 5d graph shows the compressive strength of concrete with 2% hybrid fiber.

### 3.4. Split Tensile Strength Test

At 7 and 28 days, the split tensile strength of the concrete specimens was determined.

**Figure 6:**
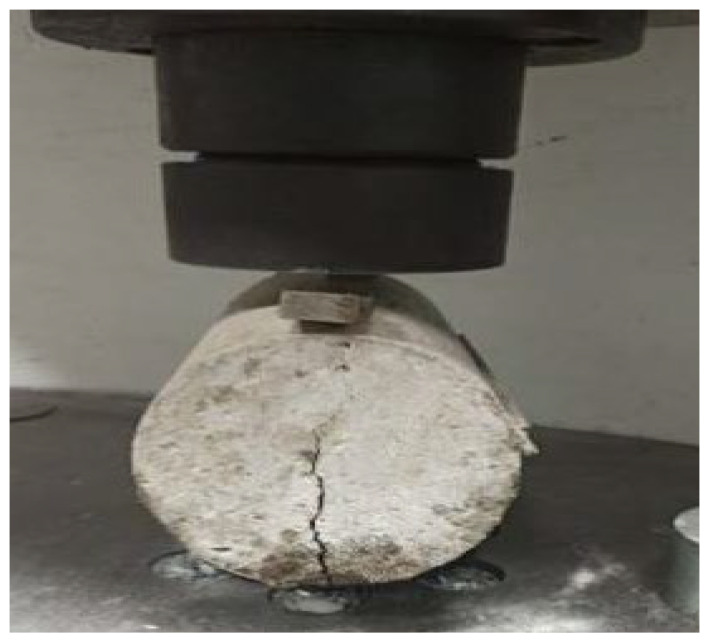
Split tensile test of the cylinder.

Table 6 summarizes the average test results of the split tensile strength in their specified curing periods of 7 and 28 days. Figure 6 shows the split tensile test of the cylinder specimen.

### 3.5. Flexural Strength Test

**Figure 7:**
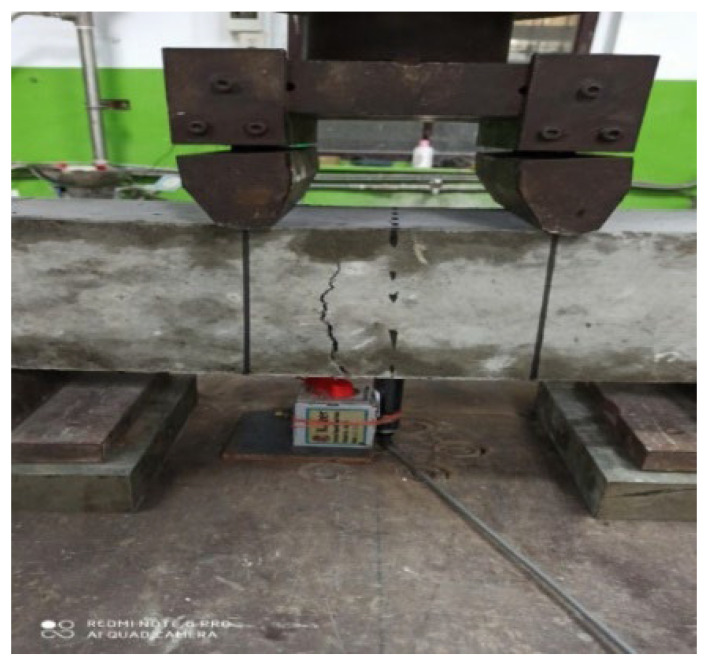
Flexural test of beam specimen (four-point loading).

The concrete specimens’ flexural strength was measured after 7 and 28 days. Table 6 summarizes the average flexural strength test results in their specified curing periods of 7 and 28 days. Figure 7 shows the flexural test of the beam specimen.

Finally, we concluded that adding a certain percentage of fiber in concrete increased the specimen’s compressive, tensile, and flexural strength since the randomly oriented fibers arrest a microcracking mechanism and limit crack propagation, thus improving stability and elasticity.

### 3.6. Relationship between Compressive Strength (fc’) Test and Modulus of Elasticity Test

According to ACI 318-19, the modulus of elasticity was calculated by the following formulas:

$$\begin{array}{cc} \; & \mathrm{Ec}=0.043\times {\mathrm{Wc}}^{1.5}\times \surd {\mathrm{fc}}^{\prime }\;(\mathrm{in}\;\mathrm{MPa})\;\ldots \ldots \ldots \mathrm{Lightweight}\;\mathrm{Concrete}\;\\ \; & \mathrm{Ec}=4700\times \surd {\mathrm{fc}}^{\prime }\;(\mathrm{in}\;\mathrm{MPa})\;\ldots \ldots \ldots \ldots \ldots \;\mathrm{Normal}\;\mathrm{Concrete}\; \end{array}$$

**Figure 8:**
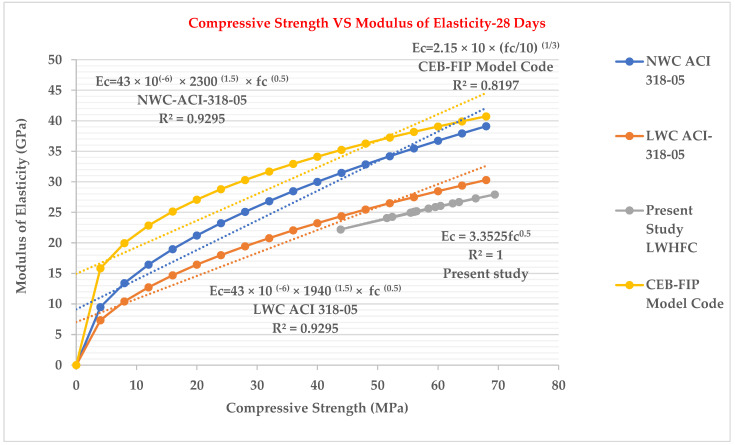
Relation between compressive strength and modulus of elasticity.

Figure 8 depicts that the cylinder compressive strength and modulus of elasticity of LWHFRC are closely related. The relationship was discovered to be as follows:

Ec = 3.3525 fc^0.5^

R² = 1

We concluded from Figure 8 that our current study’s LWHFRC regression value is close to 1, and the present study graph is close to LWC ACI 318-05. The LWC graph is higher than the normal concrete graph of Ec and fc’.

### 3.7. Relationship between Flexural Strength (fcb) and Split Tensile Strength (fspt) Test

**Figure 9:**
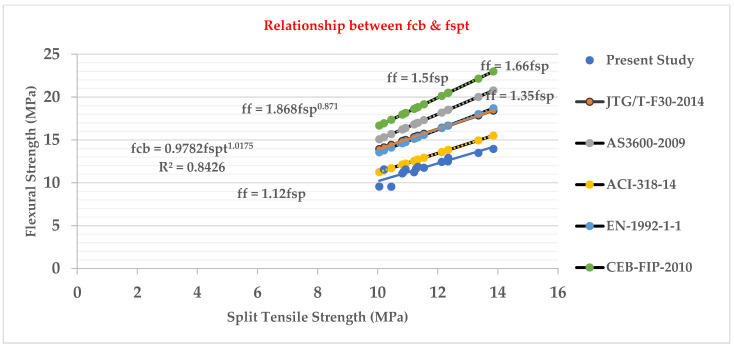
Relation between flexural strength and split tensile strength.

Figure 9 shows that the cylinder split tensile strength and beam flexural strength of LWHFRC are related. The relationship was discovered to be as follows:

fcb = 0.9782 fspt^1.0175^

R² = 0.8426

We also concluded from Figure 9 that our current study’s LWHFRC regression value is close to 0.8426, and the present study graph was similar to a normal concrete ACI 318-14 graph. However, the other normal concrete relationship graphs were higher than the current study graph [33,34,35].

### 3.8. Relationship between Split Tensile Strength (fspt) Test and Compressive Strength (fc’) Test 

**Figure 10:**
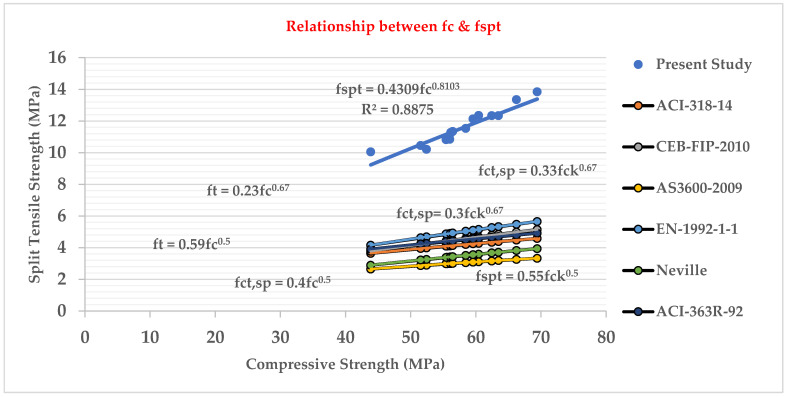
Relation between split tensile strength and compressive strength.

Figure 10 shows that the cylinder split tensile strength and compressive strength of LWHFRC are closely related. The relationship was found to be as follows:

fspt = 0.4309 fc’^0.8103^ (1)

R² = 0.8875(2)

From Figure 10, we also concluded that our present study’s LWHFRC regression value is nearly 0.8875, and the present study graph was too much higher than other graphs for normal concrete [33,34,35]. 

### 3.9. Relationship between Flexural Strength (fcb) Test and Compressive Strength (fc’) Test

**Figure 11:**
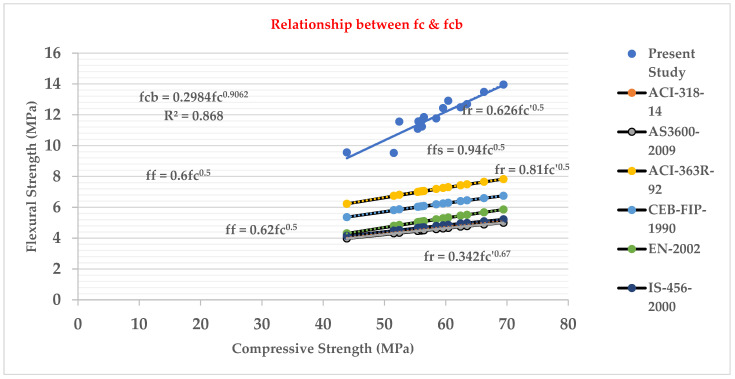
Relation between flexural strength and compressive strength.

Figure 11 shows that the beam flexural strength and compressive strength of the cylinder of LWHFRC are closely related. The relationship was found to be as follows:

fcb = 0.2984 fc’^0.9062^

R² = 0.868

From Figure 11, we also concluded that our present study’s LWHFRC regression value is nearly 0.868, and the present study graph was too much higher than other graphs for normal concrete [33,34,35].

### 3.10. Cost Analysis Report

From Table 7, Table 8 and Table 9, we concluded that if the concrete cost was increased by 16.46%, strength increased by around 55.98%; however, unit weight decreased by about 26.67%.

### 3.11. Scanning Electron Microscopy (SEM), EDS Analysis, EDS Mapping, XRD

(a) XRD Report

**Figure 12:**
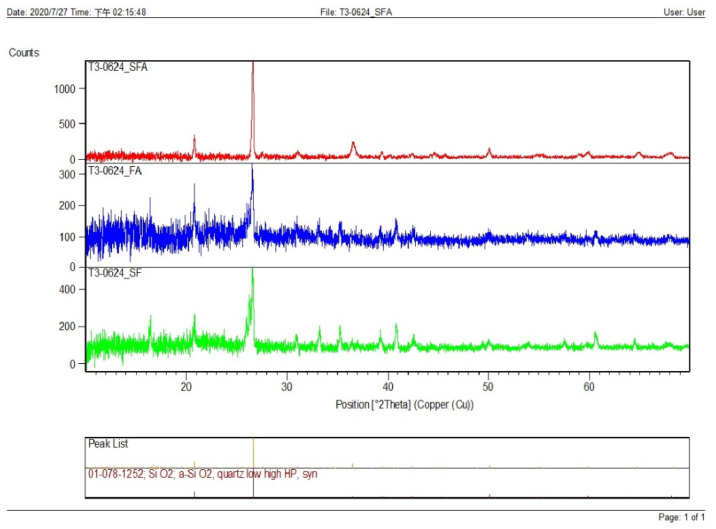
XRD report.

The SFA peak was higher than fly ash and silica fume peaks [36,37]. Figure 12 shows the XRD report.

(b) EDS Analysis

**Figure 13:**
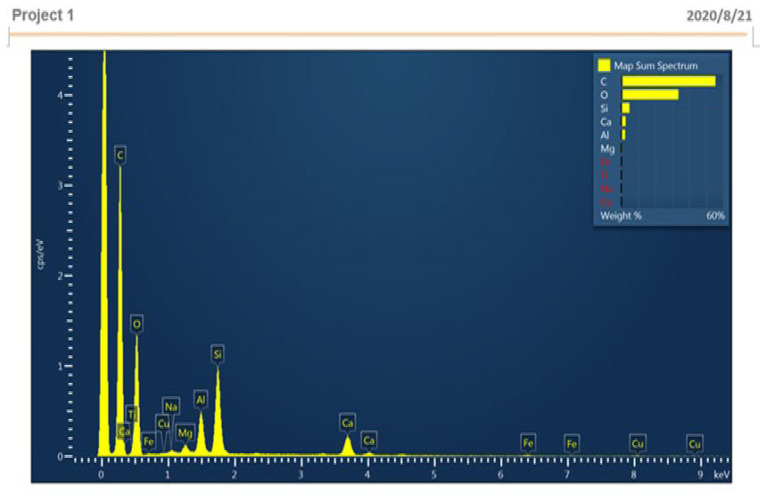
EDS analysis.

According to the EDS analysis report from Table 10, carbon was 55.39%, oxygen was 33.64%, and silicon was 4.82% [36,37]. Figure 13 shows the EDS analysis report.

(c) SEM Report

**Figure 14:**
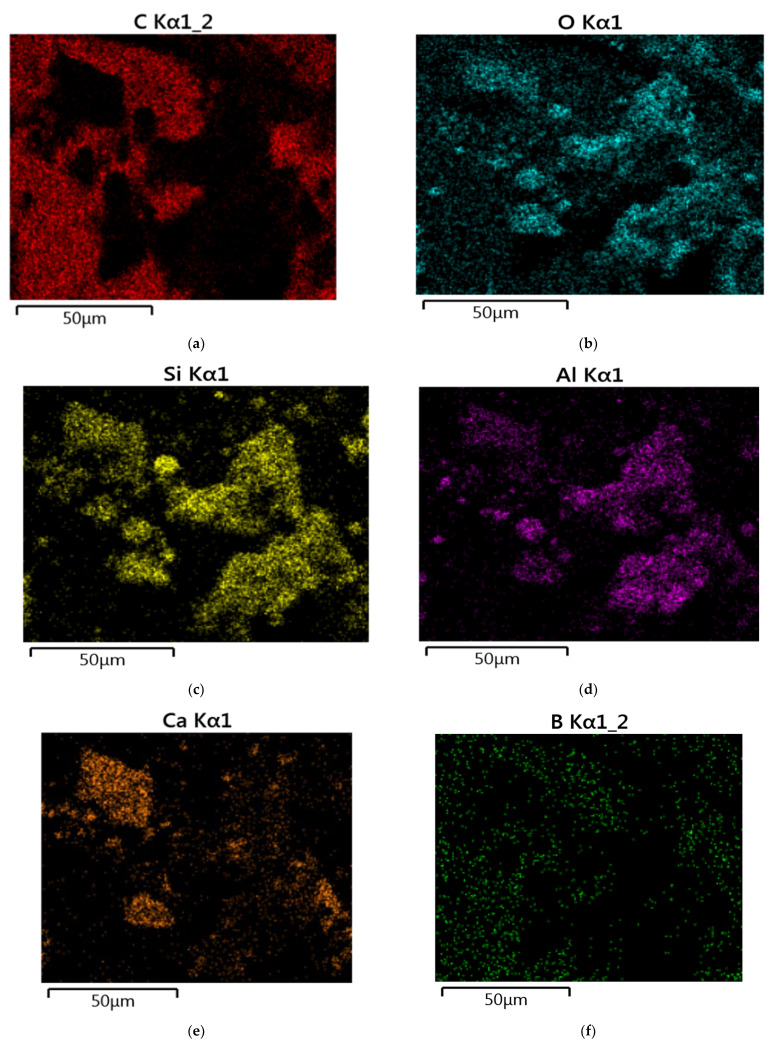
SEM image.

The SEM image in Figure 14a below showed that the carbon image is much brighter than the other image since the carbon content is greater than that of other chemicals. Similarly, in Figure 14b–f, the percentages of oxygen, silicon, aluminum calcium, and boron are simultaneously lesser; therefore, the image of oxygen, silicon, aluminum, calcium, and boron are less bright than that of carbon [36,37].

(d) SEM Report—EDS mapping

**Figure 15:**
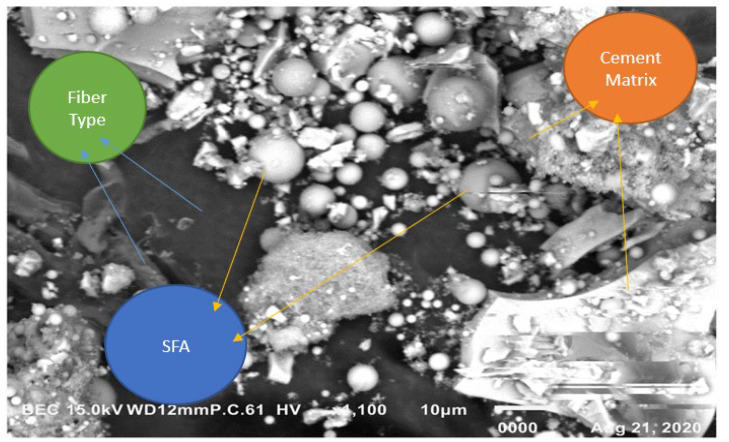
SEM image—EDS mapping on a 10 µm scale.

**Figure 16:**
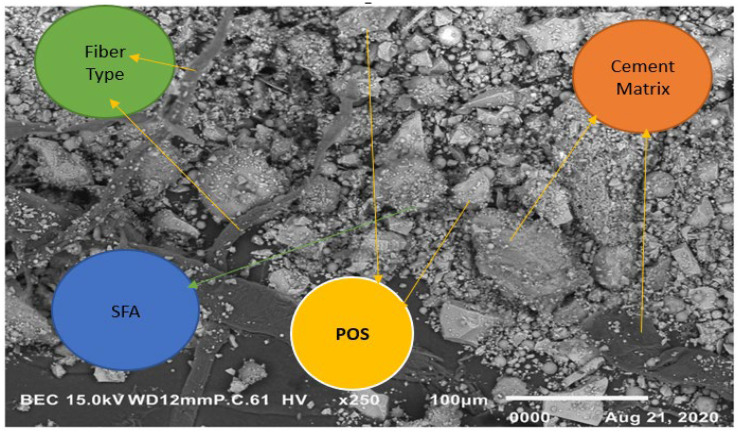
SEM image—EDS mapping on a 100 µm scale.

Morphological characteristics were found by using the JEOL-IT-100 instrument. It gives the direct result of the significant depth of field [36,37]. Figure 15 and Figure 16 show the SEM image and EDS mapping on a 10 µm scale and 100 µm scale, respectively.

### 3.12. ANOVA and Regression Analysis for Bulk Density, Water Absorption, Slump, and Compressive Strength of Lightweight Concrete and Lightweight Hybrid Fiber-Reinforced Concrete

The ANOVA and regression analysis confirmed whether our findings were significant or not. The summary of ANOVA and regression analysis are shown in Table 11. The ANOVA and regression results are shown in Table 12 and Table 13, respectively. According to the ANOVA analysis, results are significant if F statistics are greater than F critical values. ANOVA results are significant if the *p*-value is less than 0.05. The bulk density ANOVA analysis [38,39,40] for lightweight concrete was insignificant, but there was significance within SFA, POS, and LWC. Water absorption was significant between light concrete stages but not between lightweight hybrid fiber-reinforced concrete sets. Slumps were not substantial between lightweight concrete stages but were significant within the lightweight hybrid fiber-reinforced concrete stages. The difference in compressive strength for lightweight concrete was insignificant. Furthermore, there was no significance within light hybrid fiber-reinforced concrete stages.

Regression analysis revealed significant regression for bulk density, water absorption, and slump. Nonetheless, within the lightweight hybrid fiber-reinforced concrete group, only the deterioration for compressive strength was not significant (HA, HB, and HC). We concluded that regression analysis was substantial for bulk density, water absorption, and the slump in lightweight hybrid fiber-reinforced concrete. Both ANOVA and regression analysis revealed that the compressive strength of the lightweight hybrid fiber-reinforced concrete was not significant (LWHFRC). As a result, the experimental results matched the ANOVA and regression results.

## 4. Conclusions and Future Perspectives

The unit weight of concrete was around 1825 kg/m^3^ for all three stages of hybrid fiber-reinforced lightweight concrete, which met the ACI 318-19 light concrete criteria. The water binder ratio remained constant throughout all three stages at 0.30 to 0.35. (HA, HB, and HC). The average slump flow diameter is around 51 cm. The average slump height is 24.2 cm for lightweight concrete without fiber (E3). The slump value decreases gradually while the fiber percentage increases.

1. The compressive strengths (E1–E6) of LWC at 28 days were increased by 80.44%, 61.53%, 55.99%, 11.83%, 18.78%, and 4.93%, respectively, when compared to control concrete (C1).
2. Similarly, at 28 days, the compressive strengths (HA1–HA6) of LWHFRC were increased by 55.99%, 57.96%, 64.44%, 57.48%, 69.97%, and 47.50%, respectively (1% hybrid fiber). At 28 days, the compressive strengths (HB1-HB6) of LWHFRC (1.5% hybrid fiber) increased by 55.99%, 56.24%, 67.59%, 78.58%, 86.34%, and 23.42%, respectively. At the age of 28 days, the compressive strengths (HC1–HC6) of LWHFRC were increased by 55.99%, 56.24%, 58.81%, 75.63%, 95.29%, and 5.85% (-ve), respectively (2% hybrid fiber).
3. The strength of lightweight concrete without fiber increased by 55.98%, while the cost of lightweight concrete increased by approximately 16.46%, and unit weight decreased by about 26.67% compared to normal concrete without mineral admixture.
4. The significant effect of replacing 50% of the GGBS with cement played an essential role in increasing compressive strength.
5. SFA outperforms POS and NCA in compressive strength. However, the workability of SFA is lower than that of the other two aggregates. The researchers also discovered that the 90% SFA and 10% POS mixture and the 80% SFA and 20% POS mixture had higher strength and unit weight, meeting lightweight structural criteria.
6. The series of 2% steel fiber and 0.5% PVA fiber had higher compressive strength than the other percentages. The compressive strength of the 2% hybrid fiber (1.5% steel + 0.5% PVA) was higher.
7. In the flexural strength test of the beam, PVA fiber deflected more than steel fiber. As a result, the modulus of rupture of a steel fiber beam was higher.
8. The relationship between the beam flexural strength, and the cylinder split tensile strength of LWHFRC was fcb = 0.9782 fspt^1.0175,^ R^2^ = 0.8426.
9. A relationship was formed between the cylinder split tensile strength and compressive strength of LWHFRC: fspt = 0.4309 fc’^0.8103^, R^2^ = 0.8875.
10. The LWHFRC beam flexural and compressive strengths formed a relationship fcb = 0.2984 fc’^0.9062^, R^2^ = 0.868.
11. The cylinder modulus of elasticity and LWHFRC compressive strength formed a relationship E = 3.3525 fc’^0.5^, R^2^ = 1.
12. The ACI-318-19-Ec lightweight concrete equation matched the current lightweight hybrid fiber-reinforced concrete study.
13. Mineral admixtures improve the structure of the aggregate’s contact zone, resulting in a better bond between aggregates and cement paste, as demonstrated by the SEM micrograph and XRD report.
14. ANOVA and regression analysis produced the most significant results with 5 to 10% error.

## Figures and Tables

**Table 1:** Mechanical properties of materials.

Materials	Mechanical Properties
Cement	53 grades, OPC
Silica fume	Self-compaction, but SP required
Fly ash	Class F, unit weight 1380 kg/m^3^
GGBS	1000–1100 kg/m^3^ (loose), 1200–1300 kg/m^3^ (vibrated)
CA (NCA)	12 to 18 mm, an angular shape
CA (SFA)	12.5 to 18 mm, density 678 to 879 kg/m^3^, water absorption <10% for 24 h, round shape
CA (POS)	10 to 12.5 mm, density 610 to 860 kg/m^3^, water absorption < 15%, triangular shape
FA	Dune sand is 0.6 mm, and coarse sand is 4.75 mm
Water	Normal (pure)
SP	Sulfated naphthalene
Fiber—Steel	MS13/0.32 and MS16/0.2 of tensile strength 2800 MPa with an aspect ratio of 40 and 80
Fiber—PVA	6 mm and 12 mm with aspect ratio 800 and tensile strength 1500 MPa

**Table 2:** Mix design of hybrid fiber-reinforced concrete (kg/m^3^).

Mix	Cement	Silica Fume	Fly Ash	GGBS	CA (NCA)	CA (SFA)	CA (POS)	FA	Water	SP	Steel Fiber	PVA Fiber	Total
HA1	330.00	60.00	0.00	210.00	0.00	345.43	82.82	613.47	176.64	6.00	0.00	0.00	1824.35
HA2	330.00	60.00	0.00	210.00	0.00	345.43	82.82	613.47	176.64	6.00	9.12	9.12	1824.35
HA3	330.00	60.00	0.00	210.00	0.00	345.43	82.82	613.47	176.64	6.00	13.68	4.56	1824.35
HA4	330.00	60.00	0.00	210.00	0.00	345.43	82.82	613.47	176.64	6.00	4.56	13.68	1824.35
HA5	330.00	60.00	0.00	210.00	0.00	345.43	82.82	613.47	176.64	6.00	18.24	0.00	1824.35
HA6	330.00	60.00	0.00	210.00	0.00	345.43	82.82	613.47	176.64	6.00	0.00	18.24	1824.35
HB1	330.00	60.00	0.00	210.00	0.00	345.43	82.82	613.47	176.64	6.00	0.00	0.00	1824.35
HB2	330.00	60.00	0.00	210.00	0.00	345.43	82.82	613.47	176.64	6.00	9.12	18.24	1824.35
HB3	330.00	60.00	0.00	210.00	0.00	345.43	82.82	613.47	176.64	6.00	18.24	9.12	1824.35
HB4	330.00	60.00	0.00	210.00	0.00	345.43	82.82	613.47	176.64	6.00	22.80	4.56	1824.35
HB5	330.00	60.00	0.00	210.00	0.00	345.43	82.82	613.47	176.64	6.00	27.37	0.00	1824.35
HB6	330.00	60.00	0.00	210.00	0.00	345.43	82.82	613.47	176.64	6.00	0.00	27.37	1824.35
HC1	330.00	60.00	0.00	210.00	0.00	345.43	82.82	613.47	176.64	6.00	0.00	0.00	1824.35
HC2	330.00	60.00	0.00	210.00	0.00	345.43	82.82	613.47	176.64	6.00	9.12	27.37	1824.35
HC3	330.00	60.00	0.00	210.00	0.00	345.43	82.82	613.47	176.64	6.00	18.24	18.24	1824.35
HC4	330.00	60.00	0.00	210.00	0.00	345.43	82.82	613.47	176.64	6.00	27.37	9.12	1824.35
HC5	330.00	60.00	0.00	210.00	0.00	345.43	82.82	613.47	176.64	6.00	36.49	0.00	1824.35
HC6	330.00	60.00	0.00	210.00	0.00	345.43	82.82	613.47	176.64	6.00	0.00	36.49	1824.35

**Table 3:** Mix design of normal and lightweight concrete (kg/m^3^).

Mix	Cement	Silica Fume	Fly Ash	GGBS	CA (NCA)	CA (SFA)	CA (POS)	FA	Water	SP	Steel Fiber	PVA Fiber	Total
C1	600.00	0.00	0.00	0.00	1053.16	0.00	0.00	651.96	176.64	6.00	0.00	0.00	2487.76
C2	600.00	0.00	0.00	0.00	0.00	458.88	0.00	651.96	176.64	6.00	0.00	0.00	1893.48
C3	600.00	0.00	0.00	0.00	0.00	0.00	389.53	577.08	248.64	6.00	0.00	0.00	1821.25
E1	330.00	60.00	0.00	210.00	0.00	431.79	0.00	613.47	176.64	6.00	0.00	0.00	1827.89
E2	330.00	60.00	0.00	210.00	0.00	388.61	41.41	613.47	176.64	6.00	0.00	0.00	1826.12
E3	330.00	60.00	0.00	210.00	0.00	345.43	82.82	613.47	176.64	6.00	0.00	0.00	1824.35
E4	330.00	60.00	0.00	210.00	0.00	302.25	124.23	613.47	176.64	6.00	0.00	0.00	1822.58
E5	330.00	60.00	0.00	210.00	0.00	259.07	165.64	613.47	176.64	6.00	0.00	0.00	1820.81
E6	330.00	60.00	0.00	210.00	0.00	215.89	207.04	613.47	176.64	6.00	0.00	0.00	1819.04

**Table 4:** Workability test results.

**E Series (Without Fiber)—LWC**				
**Mix**	**Slump (mm)**	**Compaction Factor**	**Vee Bee Time in Seconds**	**Water Absorption %**
E1/CS	270	0.89	8	2.5
E2	255	0.87	11	3.4
E3	240	0.8	13	3.7
E4	233	0.78	15	4.6
E5	210	0.77	18	5.3
E6	190	0.75	20	5.5
**HA Series (With 1% Hybrid fiber)**				
**Mix**	**Slump (mm)**	**Compaction Factor**	**Vee Bee Time in Seconds**	**Water Absorption %**
HA1/CS	240	0.8	13	3.7
HA2	201	0.83	14	4.5
HA3	192	0.71	15	4.9
HA4	180	0.79	17	7.6
HA5	173	0.76	20	8.2
HA6	165	0.71	22	9.5
**HB Series (With 1.5% Hybrid fiber)**				
**Mix**	**Slump (mm)**	**Compaction Factor**	**Vee Bee Time in Seconds**	**Water Absorption %**
HB1/CS	240	0.8	13	3.7
HB2	210	0.74	14	4.6
HB3	194	0.71	16	5.2
HB4	180	0.68	18	5.7
HB5	162	0.69	20	11.8
HB6	154	0.64	23	12.4
**HC Series (With 2% Hybrid fiber)**				
**Mix**	**Slump (mm)**	**Compaction Factor**	**Vee Bee Time in Seconds**	**Water Absorption %**
HC1/CS	240	0.8	13	3.7
HC2	206	0.87	11	2
HC3	192	0.8	13	3.5
HC4	180	0.78	15	4
HC5	168	0.65	21	6.2
HC6	140	0.67	23	13.7

**Table 5:** A mix of unit weight ≤ 1825 kg/m^3^ fc’(MPa) without fiber.

Mix	7 Days		28 Days		% Increase Strength	
	fc’	σ	fc’	σ	7 Days	28 Days
E1	43.37	5.25	64.15	3.26	72.66	80.44
E2	38.43	5.41	57.43	5.43	52.97	61.53
E3	37.85	3.09	55.45	4.82	50.69	55.99
E4	28.52	5.24	39.75	3.5	13.53	11.83
E5	25.93	4.07	42.23	2.77	3.24	18.78
E6	22.89	3.03	37.30	2.05	−8.88	4.93
**Mix of 1% Hybrid Fiber fc’ (MPa)**						
**Mix**	**7 Days**		**28 Days**		**% Increase Strength**	
	**fc’**	**σ**	**fc’**	**σ**	**7 Days**	**28 Days**
HA1	37.85	1.77	55.45	3.73	50.69	55.99
HA2	35.43	2.95	56.16	7.60	41.02	57.96
HA3	42.58	9.59	58.46	7.56	69.50	64.44
HA4	39.75	4.75	55.98	1.80	58.26	57.48
HA5	42.49	3.93	60.43	4.96	69.14	69.97
HA6	37.15	2.53	52.44	2.41	47.87	47.50
**Mix of 1.5% Hybrid Fiber fc’ (MPa)**						
**Mix**	**7 Days**		**28 Days**		**% Increase Strength**	
	**fc’**	**σ**	**fc’**	**σ**	**7 Days**	**28 Days**
HB1	37.85	2.14	55.45	2.90	50.69	55.99
HB2	37.43	3.78	55.55	3.81	48.98	56.24
HB3	40.85	5.11	59.58	4.20	62.63	67.59
HB4	43.28	4.53	63.49	2.20	72.29	78.58
HB5	43.58	2.35	66.25	1.57	73.47	86.34
HB6	27.42	3.85	43.88	7.60	9.17	23.42
**Mix of 2% Hybrid Fiber fc’ (MPa)**						
**Mix**	**7 Days**		**28 Days**		**% Increase Strength**	
	**fc’**	**σ**	**fc’**	**σ**	**7 Days**	**28 Days**
HC1	37.85	2.25	55.45	2.84	50.69	55.99
HC2	37.43	3.17	55.55	2.69	49.00	56.24
HC3	38.62	2.28	56.46	4.38	53.76	58.81
HC4	41.87	3.93	62.44	4.92	66.68	75.63
HC5	48.66	8.27	69.43	3.86	93.70	95.29
HC6	22.43	2.13	33.47	2.17	−10.72	−5.85
C1—Control Specimen fc’ = 25.12 MPa (7 Days) and 35.55 MPa (28 Days)						

**Table 6:** Experimental results for fc’, fcb, fspt, and Ec.

Mix Stage	7 Days				28 Days			
	fc’ (MPa)	fcb (MPa)	fspt (MPa)	Ec (GPa)	fc’ (MPa)	fcb (MPa)	fspt (MPa)	Ec (GPa)
HA1	37.85	7.81	7.59	21.96	55.45	11.09	10.82	24.96
HA2	35.43	8.12	7.95	22.15	56.15	11.65	11.28	25.12
HA3	42.58	8.15	8.16	22.54	58.45	11.76	11.54	25.63
HA4	39.75	7.84	7.68	22.01	55.98	11.23	10.85	25.08
HA5	42.49	9.23	8.71	23.12	60.42	12.91	12.35	26.06
HA6	37.15	8.11	7.10	21.32	52.43	11.56	10.21	24.27
HB1	37.85	7.81	7.59	22.93	55.45	11.09	10.82	24.96
HB2	37.43	8.05	7.65	21.97	55.54	11.57	10.93	24.98
HB3	40.85	8.42	8.49	22.77	59.57	12.43	12.13	25.87
HB4	43.28	8.72	8.64	23.53	63.48	12.69	12.34	26.71
HB5	43.58	9.64	9.62	23.95	66.24	13.48	13.35	27.28
HB6	27.42	6.72	7.04	19.47	43.87	9.56	10.05	22.20
HC1	37.85	7.81	7.59	21.93	55.45	11.09	10.82	24.96
HC2	37.43	7.82	7.87	22.10	56.12	11.23	11.21	25.11
HC3	38.62	8.25	7.96	22.13	56.45	11.85	11.34	25.19
HC4	41.87	8.63	8.67	23.27	62.43	12.49	12.34	26.49
HC5	48.66	9.71	9.84	24.50	69.42	13.96	13.85	27.93
HC6	22.43	6.75	7.22	21.20	51.54	9.53	10.45	24.07

**Table 7:** C-1-100%—normal concrete in NTD.

Material	Unit Volume (m^3^)	Quantity (kg/m^3^)	Price/Ton	Total Cost (NTD)
Cement	0.19	600.00	5500.00	3300.00
Sand (FA)	0.25	651.96	450.00	293.38
NCA	0.38	1053.17	450.00	473.92
SFA	0.00	0.00	0.00	0.00
POS	0.00	0.00	0.00	0.00
Silica Fume	0.00	0.00	0.00	0.00
GGBS	0.00	0.00	0.00	0.00
Fly Ash	0.00	0.00	0.00	0.00
SP	0.01	6.00	0.00	0.00
Water	0.18	176.64	100.00	17.66
Total Quantity		2487.76		4084.97

**Table 8:** E-3 lightweight high strength concrete (80% SFA + 20% POS) in NTD.

Material	Unit Volume (m^3^)	Quantity (kg/m^3^)	Price/Ton	Total Cost (NTD)
Cement	0.10	330.00	5500.00	1815.00
Sand (FA)	0.23	613.47	450.00	276.06
NCA	0.00	0.00	450.00	0.00
SFA	0.28	345.43	350.00	120.90
POS	0.07	82.82	5600.00	463.79
Silica Fume	0.02	60.00	30000.00	1800.00
GGBS	0.09	210.00	1200.00	252.00
Fly Ash	0.00	0.00	800.00	0.00
SP	0.01	6.00	2000.00	12.00
Water	0.18	176.64	100.00	17.66
Total Quantity		1824.36		4757.42
NCA—normal coarse aggregate, NTD—New Taiwan Dollar				

**Table 9:** Cost comparison results in NTD (New Taiwan dollar).

Mix	Unit Weight (kg/m^3^)	Cost (NTD)	Fc’ (MPa)	Increment Cost	Increment Strength	Decrease Unit Weight
C1 (NC)	2487.76	4084.97	35.55	16.46%	55.98%	26.67%
E3 (LWC)	1824.36	4757.42	55.45			

**Table 10:** EDS Analysis Report.

Elements	Weight %
C	55.39
O	33.64
Na	0.18
Mg	0.43
AL	2.27
Si	4.82
Ca	2.6
Ti	0.26
Fe	0.33
Cu	0.08
Total	100

**Table 11:** Summary of ANOVA analysis—LWHFRC.

	Bulk Density	Water Absorption	Slump	Compressive Strength
Row (Between Group)	Insignificant	Significant	Significant	Insignificant
Column (Within Group)	Significant	Insignificant	Insignificant	Insignificant
Summary Of Regression Analysis—LWHFRC				
	Bulk Density	Water Absorption	Slump	Compressive Strength
Column (Within Group)	Significant	Significant	Significant	Insignificant
Column (Within Group)	Significant	Significant	Significant	Insignificant
Column (Within Group)	Significant	Significant	Significant	Insignificant

**Table 12:** ANOVA for bulk density—E series (POS, SFA, LWC).

Source of Variation	SS	DF	MS	F	*p*-Value	F Crit
Rows (Between Group)	73.06899	5	14.6138	0.002334	0.999999	3.32585
Columns (Within Group)	10,511,267	2	5,255,633	839.347	7.28 × 10^−12^	4.10281
Error	62,615.74	10	6261.574			
Total	10,573,956	17				
**ANOVA for Water Absorption—LWHFRC**						
**Source of Variation**	**SS**	**DF**	**MS**	**F**	***p*-value**	**F crit**
Rows (Between Group)	161.3783	5	32.27567	11.13594	0.000783	3.325835
Columns (Within Group)	8.843333	2	4.421667	1.525589	0.26409	4.102821
Error	28.98333	10	2.898333			
Total	199.205	17				
**ANOVA for Slump**						
**Source of Variation**	**SS**	**DF**	**MS**	**F**	***p*-value**	**F crit**
Rows	14,160.5	5	2832.1	77.45032	1.15 × 10^−7^	3.325835
Columns	52.33333	2	26.16667	0.715588	0.512325	4.102821
Error	365.6667	10	36.56667			
Total	14,578.5	17				
**ANOVA for fc’—Lightweight Hybrid Fiber-Reinforced Concrete**						
**Source of Variation**	**SS**	**DF**	**MS**	**F**	***p*** **-value**	**F crit**
Rows (Between Group)	826.0872	5	165.2174	6.623388	0.005707	3.325835
Columns (Within Group)	10.83689	2	5.418447	0.21722	0.808452	4.102821
Error	249.4455	10	24.94455			
Total	1086.37	17				

**Table 13:** Regression for bulk density.

Factors	R Square	Adjusted R Square	DF	F	Significance F
POS	1	1	1	5.94355 × 10^32^	1.69848 × 10^−65^
SFA	1	1	1	3.11206 × 10^31^	6.19518 × 10^−63^
LWC	1	1	1	1.9908 × 10^27^	1.51389 × 10^−54^
**Regression for Water Absorption**					
HA	0.953685964	0.942107455	1	82.36690647	0.000817123
HB	0.836947333	0.796184166	1	20.53195076	0.010567519
HC	0.635757264	0.544696581	1	6.981687786	0.057442761
**Regression for Slump**					
HA	0.875740011	0.844675014	1	28.19057099	0.006048326
HB	0.969230769	0.969230769	1	126	0.000358735
HC	0.963329455	0.954161818	1	105.0793708	0.000510568
**Regression for Compressive Strength**					
HA	0.008658483	−0.239176896	1	0.034936428	0.860826386
HB	0.022056558	−0.222429303	1	0.090216087	0.778866104
HC	0.076118739	−0.154851577	1	0.3295607	0.596656068

## Data Availability

Not applicable.
